# Triple Network Neuroplasticity and Perinatal Mood Symptoms from Preconception to Postpartum: A Pilot Study

**DOI:** 10.3390/jcm15155766

**Published:** 2026-07-23

**Authors:** Sharon Florentin, Atira Bick, Moriah Azoulay, Adi Klein, Amit Klein, Shiri Ben-David, Neta Elbaz Hemo, Noa Yakirevich-Amir, Catherine Monk, Netta Levin, Inbal Reuveni

**Affiliations:** 1 Faculty of Medicine, Tel Aviv University, Tel Aviv 6997801, Israel; inbalr@tlvmc.gov.il; 2 Psychiatry Division, Tel Aviv Sourasky Medical Center, Tel Aviv 6423906, Israel; 3 Department of Psychiatry, Hadassah Hebrew University Medical Center, Jerusalem 9112001, Israel; neta.elbaz@mail.huji.ac.il (N.E.H.); noay@hadassah.org.il (N.Y.-A.); 4 Department of Neurology, Hadassah Hebrew University Medical Center, Jerusalem 9112001, Israel; bicka@hadassah.org.il (A.B.); netta@hadassah.org.il (N.L.); 5 Department of Psychology, The Hebrew University of Jerusalem, Jerusalem 9190501, Israel; moria.azoulay@mail.huji.ac.il (M.A.); adi.klein@mail.huji.ac.il (A.K.); shiri.ben-david@mail.huji.ac.il (S.B.-D.); 6 Department of Psychology, Hadassah Hebrew University Medical Center, Jerusalem 9112001, Israel; 7 Department of Obstetrics and Gynecology, Hadassah Medical Center, Jerusalem 9112001, Israel; 8 Department of Obstetrics and Gynecology, Vagelos College of Physicians and Surgeons, Columbia University Irving Medical Center, New York, NY 10032, USA; cem31@cumc.columbia.edu; 9 New York State Psychiatric Institute, New York, NY 10032, USA

**Keywords:** perinatal mood and anxiety disorder (PMAD), fMRI, resting state, perinatal neuroplasticity

## Abstract

**Background:** Perinatal brain network alterations, including changes in resting-state functional connectivity (rsFC), may increase vulnerability for perinatal mood and anxiety disorders (PMAD). The Fronto-Parietal Network (FPN), the Default-Mode Network (DMN), and the Salience Network (SN) are interconnected networks implicated in mood disorders. However, these networks’ role in perinatal neuroplasticity and PMAD remains unclear. **Methods:** This pilot study prospectively followed twenty-two women from preconception to postpartum. Participants completed anxiety and depression questionnaires and underwent fMRI scans at preconception and postpartum. The FPN, DMN, and SN were defined and average connectivity of each network was determined. Differences in connectivity from preconception to postpartum within each network and to each network at the whole-brain level were computed. **Results:** While connectivity within the FPN, DMN, and SN did not show significant changes from preconception to postpartum, we found increased rsFC from the left anterior prefrontal cortex to the DMN, and decreased rsFC from the right ventral posterior cingulate cortex to the FPN, from preconception to postpartum. Bilateral supplementary motor areas (SMAs) showed increased connectivity to the FPN from preconception to postpartum, and this change in connectivity was negatively correlated with anxiety scores during pregnancy. Right SMA connectivity to the FPN at preconception showed positive association with postpartum depression scores (*p* < 0.05 for all comparisons). **Conclusions:** RsFC perinatal-related changes were demonstrated in cognitive and motor regions connected to the DMN and FPN. Among women with perinatal depressive and anxiety symptoms, changes in SMA rsFC to the FPN from preconception to postpartum were observed, indicating possible roles in PMAD.

## 1. Introduction

Perinatal mood and anxiety disorders (PMADs) affect one in five pregnant and postpartum women, with long-term consequences for both mother and child [1,2,3,4,5,6,7,8,9]. Common risk factors include a history of depression, poor social support, stressful life events, and pregnancy complications [10,11,12,13]. However, little is known about the neurobiological underpinnings of PMAD.

### 1.1. Neuroplasticity of the Maternal Brain

Brain plasticity during pregnancy is thought to be part of an adaptive process preparing mothers for parenthood. Studies have shown structural brain changes in women, including a reduction in gray matter volume, from preconception to the postpartum period [14], similar to those shown in adolescent females, suggesting hormonal fluctuations may affect neuroplasticity [15]. The reduction in grey matter is seen during late pregnancy [16] and partially recovers during the postpartum period [17,18,19]. Postpartum increase in axon density in white matter tracts across pregnancy has also been reported [20]. A recent study found that mother’s well-being mediates the relationship between postpartum global grey matter volume recovery and maternal postpartum attachment, while depression and perceived stress scores did not mediate this relationship [19]. Perinatal functional neuroplasticity, as indicated in task-based and resting-state functional brain patterns, occurs in brain regions involved in maternal roles, including the Reward System [21,22], Salience Network (SN) [23], and the Default Mode Network (DMN) [14,24,25], which are linked to maternal attachment [26] and caregiving [27,28].

### 1.2. The Triple Network Model

The triple network model includes three functional networks: the fronto-parietal network (FPN) composed mainly of the dorsolateral prefrontal cortex (DLPFC) and posterior parietal cortex (PPC), involved in sustained attention, complex problem-solving and working memory [29,30,31]; the DMN, consisting of the medial prefrontal cortex (mPFC), posterior cingulate cortex (PCC), and angular gyrus, involved in emotional processing, self-referential activity, the recollection of prior experiences, and social cognition [32,33,34]; and the SN consisting of the anterior insula and dorsal anterior cingulate cortex (dACC), responsible for salience detection and directing behavior to the most pertinent actions [35,36,37]. The FPN activity was found to be negatively correlated with the DMN [38]. The SN is thought to mediate the engagement of the FPN and disengagement of the DMN and vice versa, and the dynamic interplay between externally and internally focused attention and cognitive-affective processing [36,39,40]. Hormonal fluctuations impact brain network organization, particularly within the DMN during ovulation [41]. Longitudinal studies regarding the triple network in the perinatal period are scarce. A recent longitudinal study evaluating rsFC at preconception and postpartum, which assessed Yeo’s 7-large-scale functional networks changes (including the DMN and FPN) [42], did not find significant changes within network or network segregation [19]. However, gray matter reductions in pregnant women are most evident and steepest in DMN and FPN regions, highlighting their role in perinatal plasticity [16,19,43]. However, whether changes in perinatal neuroplasticity contribute to vulnerability for PMAD has rarely been studied.

### 1.3. Resting-State Functional Connectivity and PMAD

Neural circuit alterations, including changes in resting-state functional connectivity (rsFC), during the postpartum period may increase vulnerability to PMAD [44,45]. Studies show reduced connectivity between the ACC, bilateral amygdalae, hippocampus, and DLPFC and other corticocortical and corticolimbic regions in women with postpartum depression (PPD) [44], resembling patterns in non-perinatal depression [46,47,48]. Another study revealed disrupted connectivity between the right amygdala and the PCC among PPD participants [45]. PPD often co-occurs with anxiety (PPD-A) [49,50], and reduced rsFC was shown between dorsomedial prefrontal cortex (dmPFC) and left ventral striatum (VS.L) in both PPD and PPD-A women [51]. PPD revealed specific changes in rsFC between right lingual gyrus and dmPFC, and also between the right angular gyrus and left precentral gyrus, while PPD-A represented abnormal rsFC between VS.L and left ventrolateral prefrontal cortex [51].

Research on the ‘triple network’ and PMAD is limited, with most studies focusing on non-perinatal depression. These studies show altered rsFC including hypoconnectivity within the FPN, hyperconnectivity within the DMN, and hyperconnectivity between the FPN and regions of the DMN [52,53]. Identifying neural patterns linked to PMAD may enable early detection and intervention. To the best of our knowledge, this is the first study to examine whether perinatal neuroplasticity changes within the triple network and alterations in whole-brain connectivity to these networks, may contribute to vulnerability to PMAD.

Therefore, this pilot study aimed to examine (1) Changes in rsFC from preconception to postpartum within the FPN, DMN, and the SN and in the connectivity of other brain regions to these networks and (2) Associations between rsFC changes with anxiety and depression symptom severity during pregnancy and postpartum. We hypothesized that (1) rsFC within the DMN will decrease from preconception to postpartum, while rsFC within the FPN will increase, as these networks are known to be negatively correlated. (2) rsFC changes from preconception to postpartum within the FPN, DMN, and the SN, and between these networks to brain regions at the whole-brain level will be correlated with anxiety and depression symptoms reported during pregnancy and postpartum.

## 2. Materials and Methods

### 2.1. Participants

Healthy, non-pregnant, nulliparous euthymic women (ages 18–40) attempting conception without fertility treatments were recruited. Semi-structured clinical interviews ensured exclusion criteria were met. Of 298 women recruited (November 2020–November 2023), 140 met eligibility and were followed through preconception, pregnancy, and postpartum. Among 140 participants, 79 underwent preconception resting-state fMRI, and 22 who gave birth completed a second fMRI scan postpartum. No demographic or clinical differences were found between those who had two fMRI scans and the rest of the group. Group comparisons (using chi-square tests for categorical variables and independent *t*-tests for continuous variables) showed no significant differences in age (*p* = 0.826), years of education (*p* = 0.201), marital status (*p* = 0.680), or social support (*p* = 0.289). Similarly, baseline and follow-up depression and anxiety scores across preconception, pregnancy, and postpartum did not differ between the groups (all *p*-values ranging from 0.100 to 0.968). All women had completed self-report questionnaires longitudinally at five time points: preconception, 1st trimester (weeks 10–13), 2nd trimester (weeks 24–28), 3rd trimester (weeks 32–36), and postpartum (6–12 weeks after delivery). During the postpartum follow-up they also reported pregnancy complications. The study was approved by the Hadassah-Hebrew University Medical Center Ethics Committee (experiment name/number: 0301-20-HMO), and all participants provided written informed consent. All methods were performed in accordance with the relevant regulations and guidelines according to the Declaration of Helsinki.

Exclusion criteria included substance abuse in the past six months, a history of psychotic or bipolar disorders, a major depressive episode in the past year as determined by the Diagnostic Interview for Anxiety, Mood, and Obsessive Compulsive Disorders and Related Neuropsychiatric Disorders (DIAMOND) interview [54], current psychiatric care, medical conditions posing pregnancy risks, fertility treatment needs, medication use (except perinatal vitamins), and MRI contraindications (implanted ferromagnetic devices, pacemakers, claustrophobia, or a positive pregnancy test on the day of the MRI or prior to it).

### 2.2. Clinical Measures

Depression symptoms were measured using the Patient Health Questionnaire (PHQ-9), a validated self-report tool measuring depression severity [55]. Depression scores are typically interpreted as follows: 0–4 (none/minimal), 5–9 (mild), 10–14 (moderate), 15–19 (moderately severe), and 20–27 (severe). Participants completed the questionnaire at five time points (preconception, during each trimester, and after delivery).

Anxiety symptoms were measured using the General Anxiety Disorder Questionnaire (GAD-7) [56]. Anxiety scores are typically interpreted as follows: 0–4 (minimal), 5–9 (mild), 10–14 (moderate), 15–21 (severe). Participants completed the questionnaire at five time points (preconception, during each trimester, and after delivery).

There is vast literature demonstrating that social support is a prominent risk factor for PMAD [11,12,13]. Therefore, levels of social support were evaluated at preconception using the Social Support and Social Undermining Questionnaire [57,58].

### 2.3. fMRI Data Acquisition

MRI scans were conducted using a 3T Siemens Skyra scanner (Siemens Healthineers, Forchheim, Germany) at the Edmond & Lily Safra Center for Brain Sciences Neuroimaging unit. Anatomical imaging included high-resolution T1-weighted sequences (TE: min, TR: 2300 ms, voxel size: 1 × 1 × 1 mm). Resting-state BOLD fMRI acquired using gradient-echo echo-planar imaging (TR: 1000 ms, TE: 32 ms, flip angle: 62°, in-plane resolution: 2.5 × 2.5 mm, matrix: 76 × 76, FOV: 190 × 190 mm, 52 slices, 2.5 mm each, 360 repetitions).

Preprocessing was done using BrainVoyager QX software package 3.6.2 (Brain Innovation) standard preprocessing streamline. This included head motion correction, slice timing correction, and high-pass filtering (3 cycles/total scan time) to enhance signal quality. Average motion was small (0.53 mm), and no significant difference in motion size was found between visits (*p* = 0.26). Individual evaluation of motion in subjects identified only one subject with significant motion (in first visit: 4.7 mm, in second visit 0.69. For all other subjects motion was smaller than 1.17 mm). Connectivity maps for this subject were inspected, and as networks seemed adequate and beta values were within range, we did not remove her from analysis. No spatial smoothing was applied, neither was physiological noise collected and removed from data. Anatomical and functional images were co-registered and normalized to Montreal Neurological Institute (MNI) space. [59].

### 2.4. Resting-State Functional Connectivity Analysis

Seed point-based rsFC was used to identify the DMN, FPN, and SN. The seed’s region of interest (ROI) was chosen by creating a sphere (8 MNI coordinate in diameter) around the predefined DMN, FPN, and SN MNI coordinates [42]. For the FPN and SN four bilateral regions of interest (ROIs) were selected, and for the DMN eight bilateral ROIs. The mean values of the time series of the ROIs were extracted for each subject before and after pregnancy and used as a predictor for whole-brain general linear model (GLM) analysis to obtain the preconception and postpartum individual connectivity maps for each of the three networks. For each network a group map was obtained, and beta parameters were extracted from the network regions (*p* < 0.05) for each subject for further analysis. Individual network maps were used for whole-brain connectivity analysis to measure functional connectivity within and to the three functional brain networks. Connectivity differences from preconception to postpartum were analyzed using paired *t*-tests, with Monte Carlo correction for multiple comparisons (Full Width at Half Maximum = 1.136 functional voxel. 1000 iterations. *p* < 0.05, resulting in cluster size 37 mm). Beta parameters from significant regions were extracted for further statistical analysis alongside demographic and clinical data. All functional data analyses were conducted using the BrainVoyager QX software package (Brain Innovation).

### 2.5. Statistical Analysis

Repeated-measures ANCOVA tracked changes over time in anxiety and depression scores, adjusting for age, social support, and pregnancy complications (excluding major depressive disorder (MDD) history, as none had prior diagnoses). ANCOVA examined the associations between preconception scan and anxiety/depression scores during pregnancy and postpartum, as well as between brain connectivity changes and anxiety/depression scores, controlling for the covariates mentioned above. Delivery-to-scan time was added as a covariate in all ANCOVAs that included connectivity changes between postpartum and preconception scans. Statistical significance was set at *p* < 0.05, analyzed using IBM SPSS Statistics 26.0.

## 3. Results

Demographic and clinical characteristics of the study group are presented in Table 1.

### 3.1. Pregnancy-Related Resting-State Functional Connectivity Changes

**Figure 1:**
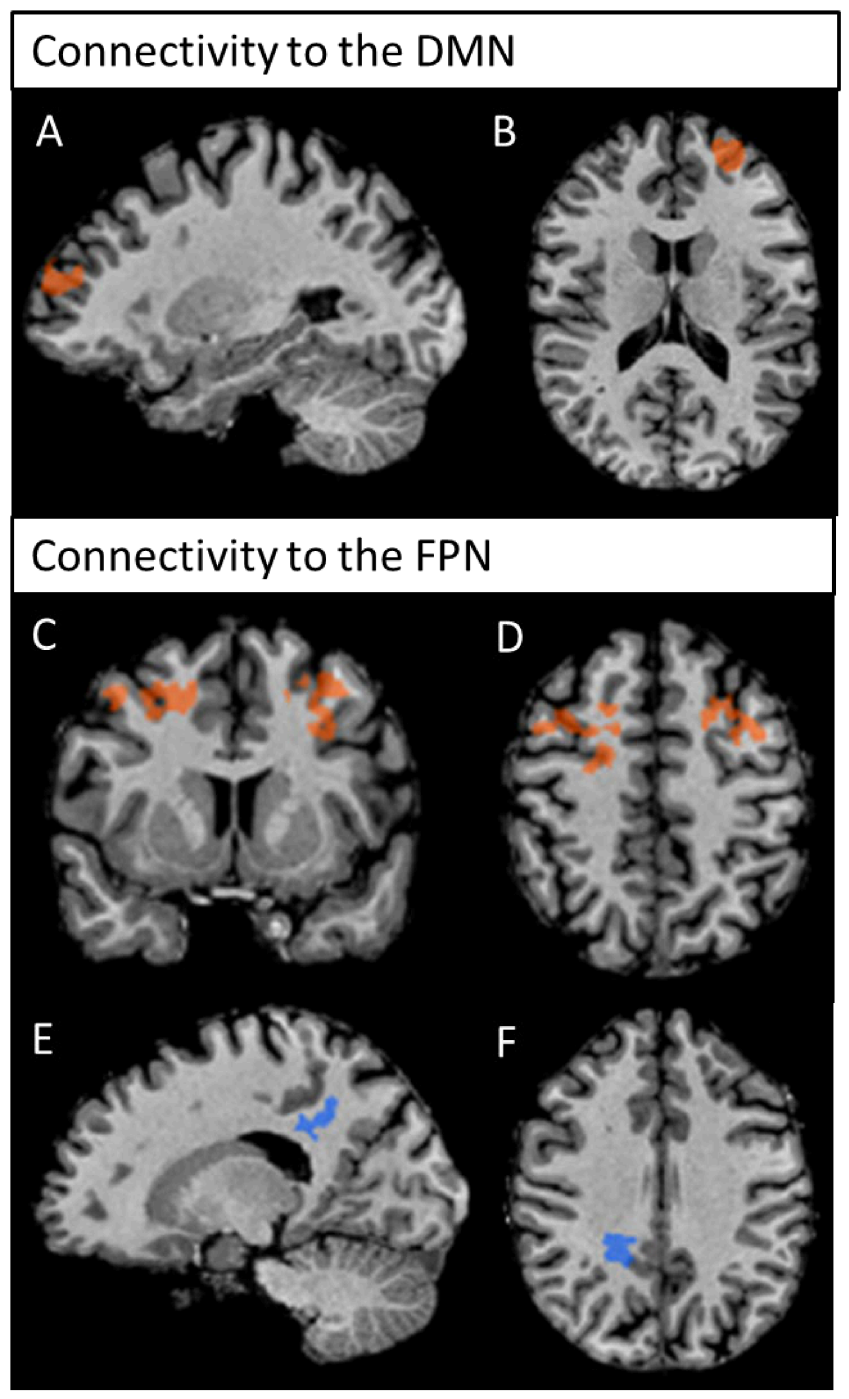
Changes in connectivity to the DMN and the FPN from preconception to postpartum. DMN= Default-Mode Network; FPN= Frontoparietal Network. (**A**)—L anterior PFC (sagittal); (**B**)—L anterior PFC (axial). (**C**)—R&L SMA (coronal); (**D**)—R&L SMA (axial). (**E**)—R ventral PCC (sagittal); (**F**)—R Ventral PCC (axial). Blue—decreased connectivity; Orange—increased connectivity.

There were no significant rsFC differences within the networks from preconception to postpartum in group network maps or whole-brain paired *t*-tests. However, whole-brain GLM analysis revealed significant changes in connectivity to the DMN and the FPN from preconception to postpartum in regions beyond these networks: increased rsFC of the left anterior PFC to the DMN, increased rsFC of the left and right supplementary motor areas (SMA) to the FPN, and decreased rsFC of the right ventral PCC to the FPN (Table 2, Figure 1). No region showed significant changes in the connectivity to the SN from preconception to postpartum.

### 3.2. Changes in Depression and Anxiety Scores from Preconception to Pregnancy and Postpartum

**Figure 2:**
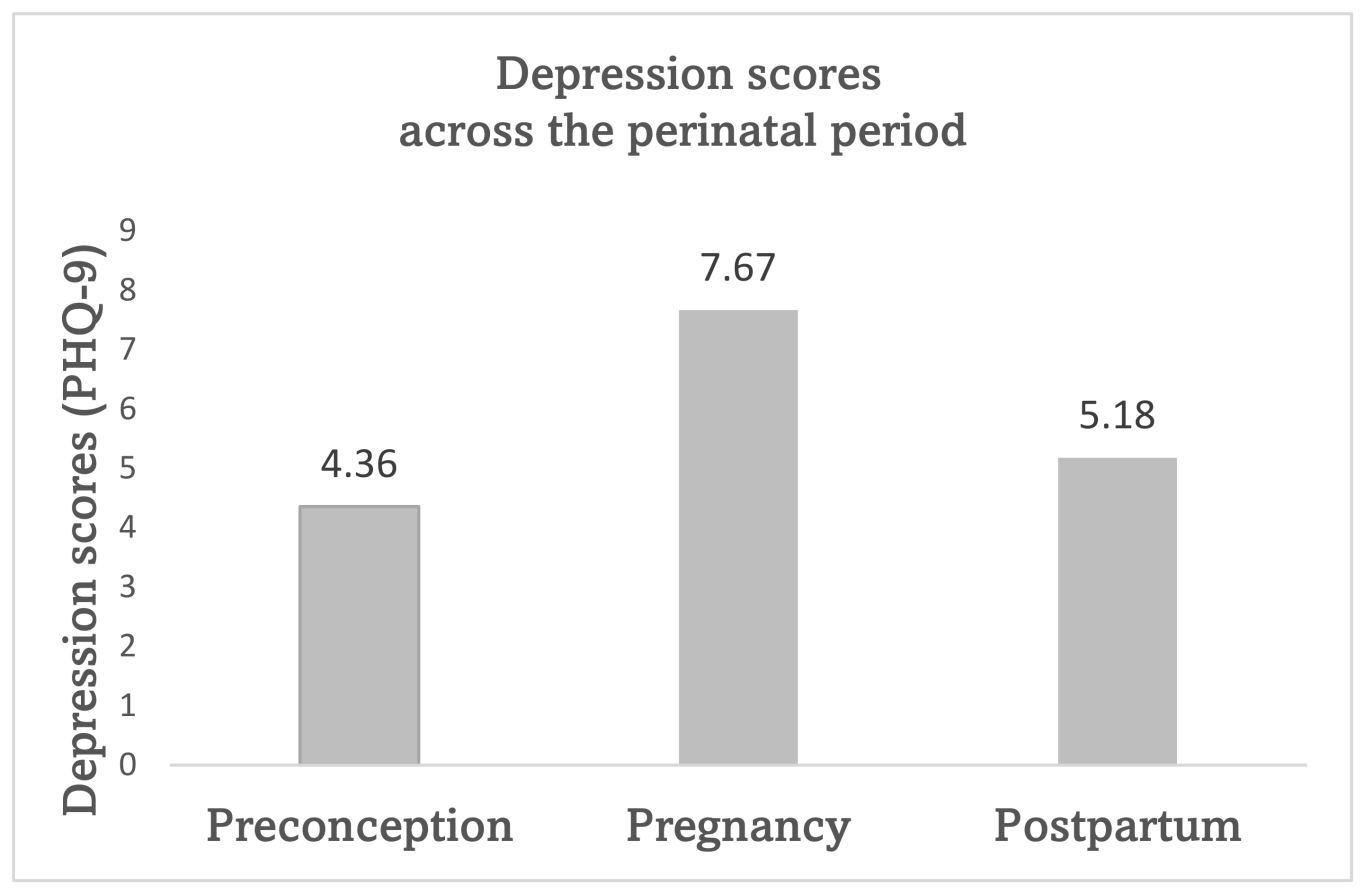
Mean depression scores during preconception, pregnancy, and postpartum. PHQ-9 = Patient Health Questionnaire.

The number of women with depression of moderate severity or higher increased from one (5%) before pregnancy, to six (27%) during pregnancy, and then declined to two (9%) during postpartum. Repeated measures ANOVA model was used to assess the changes in mean depression scores during three periods: preconception, pregnancy, and postpartum. Mean PHQ-9 scores increased from 4.36 (SD 2.63) at preconception to 7.67 (SD 3.51) during pregnancy, and then declined to 5.18 (SD 4.42) at postpartum (Figure 2). The period effect for depression scores was significant (within subject period effect: F = 12.92, *p* = 0.002). After adjusting for age, social support, and pregnancy complications, the period effect disappeared (*p* = 0.16), and pregnancy complications exhibited an independent significant effect on depression scores (F = 7.55, *p* = 0.013). Repeated measures ANOVA model for mean anxiety scores during the three periods, did not reveal any significant effect.

### 3.3. Perinatal Depressive and Anxiety Symptoms and Resting-State Functional Connectivity

**Figure 3:**
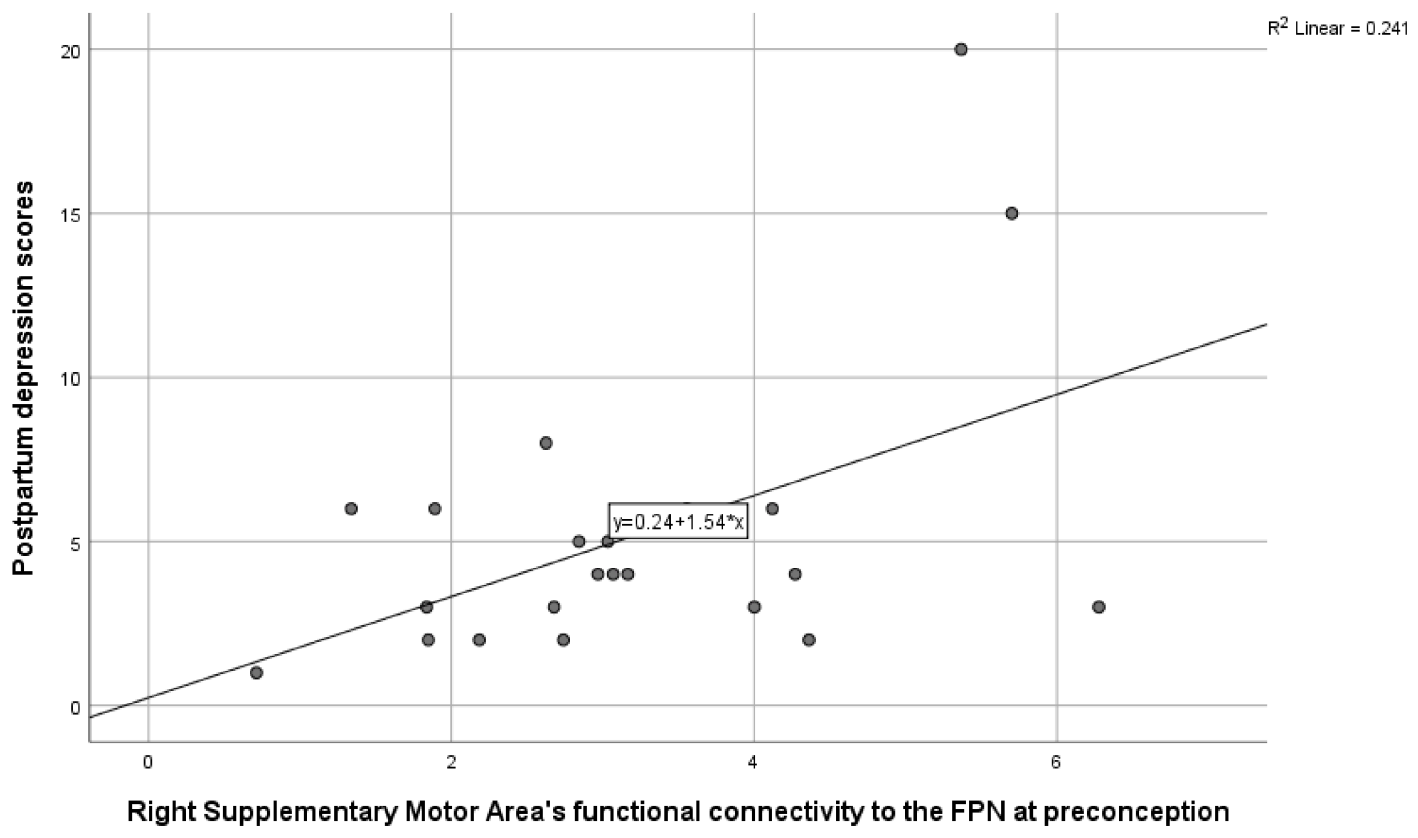
Correlation between resting-state functional connectivity of the supplementary motor area to the FPN at preconception and postpartum depression scores. No Significant correlation to depression and anxiety scores at postpartum was found for the other three regions (L anterior PFC, L SMA, R ventral PCC) that underwent significant change in rsFC following pregnancy.

The regions showing pre-to-post pregnancy changes were further evaluated to identify connections between connectivity and behavior. ANCOVA revealed that depression scores at postpartum can be predicted by rsFC at preconception: rsFC at preconception between the R SMA and the FPN was associated with higher depression score at postpartum (F = 5.88, *p* = 0.027, partial eta squared = 0.26) (Figure 3). Pregnancy complications also had significant effect (F = 6.72, *p* = 0.019, partial eta squared = 0.28) on depression score. Social support and age were included in the model as well but did not contribute to prediction. Fifty percent of the variance in the depression scores was explained by these four independent variables (r^2^ = 0.47, F = 3.77, df = 4, *p* = 0.023).

**Figure 4:**
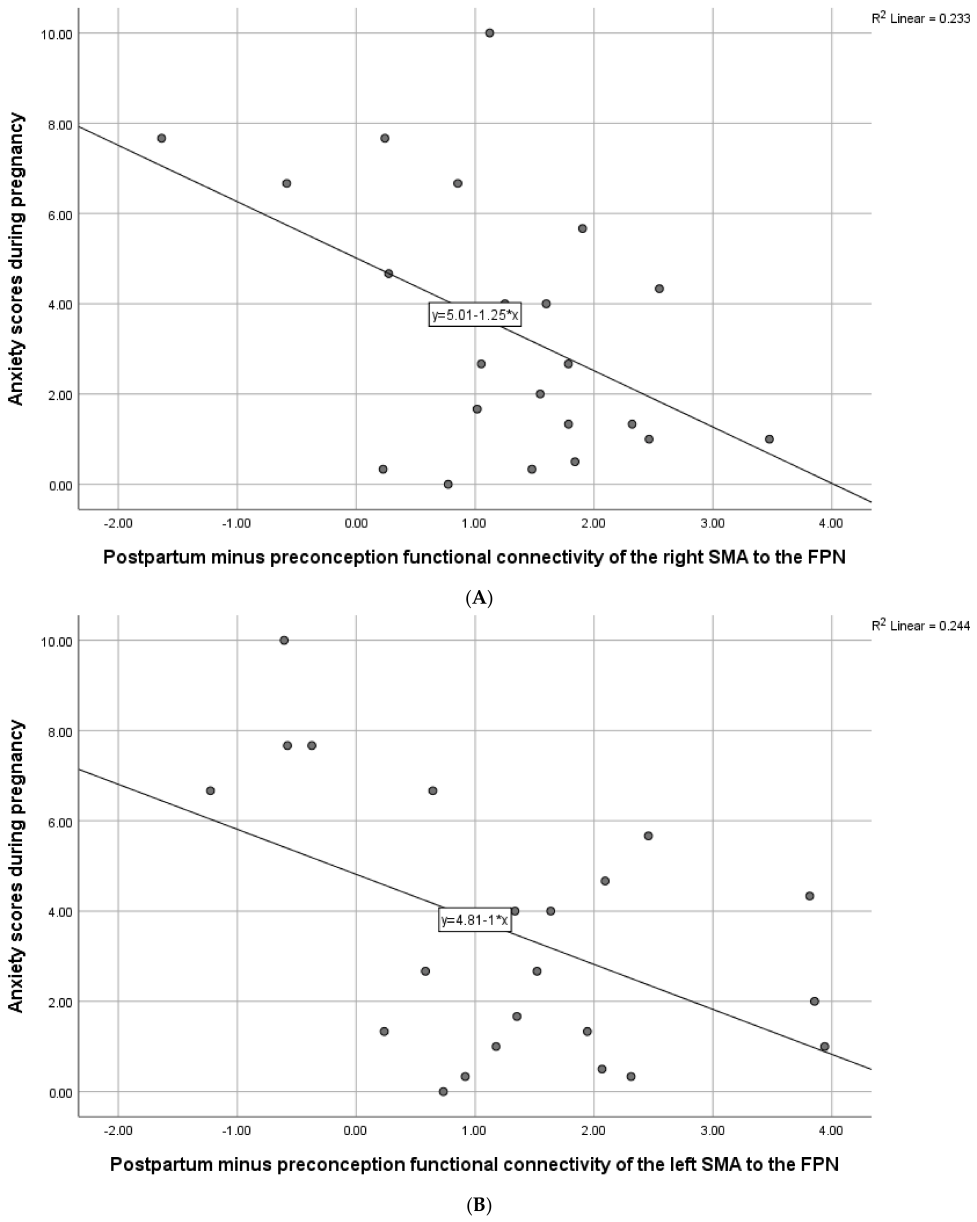
Correlation between the change in resting-state functional connectivity from preconception to postpartum of the right (**A**) and left (**B**) supplementary motor area to the FPN and anxiety scores during pregnancy.

ANCOVA modeling for anxiety scores during pregnancy (mean score of the three trimesters), using the same independent variables mentioned above, revealed that anxiety during pregnancy was negatively associated with the change from preconception to postpartum in rsFC between both the right and left SMA (each one in a separate model) and the FPN (F = 11.54, *p* = 0.003, partial eta squared = 0.40 for R SMA; F = 8.16, *p* = 0.011, partial eta squared =0.32 for L SMA). The higher the rsFC at postpartum compared to preconception, the anxiety scores were lower (Figure 4A,B). Pregnancy complications, which were included as a covariate, also were associated with anxiety scores (F = 7.51, *p* = 0.014, partial eta squared = 0.31 for R SMA; F = 6.32, *p* = 0.022, partial eta squared = 0.27 for L SMA). The independent variables accounted for 54% and 48% of the variance in the anxiety scores during pregnancy in the model with the R SMA and the L SMA respectively (r^2^ = 0.54, F = 4.98, df = 2, *p* = 0.008 for R SMA; r^2^ = 0.48, F = 3.88, df = 4, *p* = 0.02).

The other two regions (L anterior PFC, R ventral PCC) that underwent significant change in the rsFC from preconception to postpartum did not show a significant correlation between the change in the rsFC and anxiety scores during pregnancy and postpartum. Additionally, depression scores during pregnancy showed no correlation with rsFC changes.

## 4. Discussion

As far as we know, this pilot study is the first longitudinal study following women from preconception until postpartum that investigates perinatal functional neuroplasticity and its relation to perinatal depressive and anxiety symptoms by measuring changes in the rsFC of three interconnected large-scale networks and changes in whole-brain connectivity to these networks. While overall network connectivity remained stable, similar to recent evidence [19], rsFC alterations emerged in additional regions linked to the DMN and FPN, with only FPN connectivity changes correlating with perinatal depressive and anxiety symptoms.

Brain regions, specifically the L&R SMA, the R PCC, and the L anterior PFC, show changes in rsFC to the FPN and DMN from preconception to postpartum, consistent with regions previously shown to undergo a decrease in grey-matter volume and structural changes during the perinatal period [14,19,43], and may be part of the neurobiological adaptations to pregnancy and parenthood. Among these regions, the PCC, part of the DMN, is the main area known to be related to cognitive and emotional functions relevant for maternal behavior [28,45,60]. Studies show disrupted PCC–amygdala connectivity has been linked to PPD [45], and reduced DMN connectivity during working memory tasks in postpartum women [60].

Hoekzema et al. reported increased rsFC within the DMN from preconception to postpartum [43], while we have found only increased rsFC of the anterior PFC to the DMN. Differences may stem from the fMRI analyses method and demographic variations, including a younger sample in our study. However, Servin-Barthet et al. did not find any within network perinatal changes, similar to our results [19].

FPN connectivity changes, from preconception to postpartum, were observed in two main areas. The R ventral PCC (DMN) showed reduced rsFC to the FPN, aligning with its known negative correlation with the DMN [38]. The L&R SMA exhibited increased rsFC to the FPN. The FPN plays a critical role in cognitive control by facilitating sustained attention, working memory manipulation, and goal-directed behavioral adaptation [29,30,31]. Consequently, during the perinatal period, enhanced FPN function may help regulate postpartum mood fluctuations through cognitive control and task-oriented processing. Meanwhile, the SMA is involved in preparing and organizing voluntary movements, particularly those requiring precise sequencing or initiation [61]. During complex behaviors, the FPN can exert top-down control over SMA activity to modulate motor output [62]. In line with this, the observed increase in resting-state functional connectivity (rsFC) between the bilateral SMA and the FPN supports the network’s integrated role in cognitive and motor sequencing [63]. These neural adaptations could reflect the maternal motor synchronization required to dynamically respond and adapt movements to the infant’s changing needs.

We found that depression scores increased during pregnancy but decreased postpartum, possibly due to stress, hormonal, and physical changes [64], with varying trajectories across clinical phenotypes [65]. Prior research on perinatal depression trends is mixed: some report an increase in depression during postpartum [66], while others show a decline [67]. A systematic review of studies from 51 low- and middle-income countries supports a postpartum decrease [68], aligning with our results. The decline may be influenced by our study population’s characteristics—healthy women with high education, social support, and stable socioeconomic status.

R SMA connectivity at preconception positively correlated with postpartum depression scores, whereas longitudinal increases in bilateral SMA-FPN connectivity from preconception to postpartum were negatively associated with anxiety during pregnancy. This indicates that while baseline, pre-pregnancy network connectivity may serve as a vulnerability marker for future postpartum depression, the dynamic, peripartum connectivity of SMA-FPN coupling tracks active anxiety states. While this study is the first to associate SMA-FPN connectivity changes with perinatal mood symptoms, these novel data represent exploratory pathways suggesting a potential neurobiological association that requires prospective replication in larger samples.

Previous research regarding non-perinatal depression has linked psychomotor retardation to altered structural connectivity in motor pathways [69,70] and in task-based connectivity between SMA and basal ganglia [61]. Resting-state SMA blood flow has been linked to motor retardation in MDD [35,71]. Similarly, we found that the rsFC of the R SMA to the FPN at preconception had a positive correlation with postpartum depression scores. SMA involvement in premenstrual dysphoric disorder may suggest vulnerability to mood fluctuations [72,73,74]. Moreover, anxiety disorders show decreased SMA activation [75]. On the other hand, individuals with anxiety demonstrated decreased activation in the SMA after receiving psychotherapy [76]. In our study, L&R SMA connectivity to the FPN increased from preconception to postpartum, but decreased connectivity was associated with anxiety during pregnancy. This may suggest that women with anxiety during pregnancy may not exhibit the same adaptive perinatal neuroplasticity alterations as healthy women without anxiety.

The study’s main limitation is its small, relatively homogenous sample, restricting generalizability, thereby we suggest to consider this a pilot study. Significant results related to the FPN lacked clinical cognitive measures warranting future research on the correlation between brain changes and maternal cognitive functioning. Additionally, a temporal gap averaging 4 months occurred between the postpartum clinical assessments and fMRI scans, reflecting the naturalistic data collection design of the longitudinal study. Because perinatal mood, hormonal profiles, and caregiving dynamics fluctuate dynamically during this period, the imaging data may not fully reflect the clinical status at the time of behavioral testing. Specifically, clinical symptoms measured at 2 months postpartum on average may have been more pronounced than those present during the scan 4 months later, as some participants likely adjusted to the changes of early motherhood over time. Although we adjusted for ‘time from birth to scan’ in our statistical models to mitigate this variance, these findings should be interpreted cautiously, and future studies should aim for tightly synchronized sessions. Finally, current depression and anxiety measures may not fully capture perinatal emotional, cognitive, and behavioral changes, highlighting the need for comprehensive measures of the different facets of the transition to motherhood.

## 5. Conclusions

This pilot longitudinal study has revealed rsFC alterations from preconception to postpartum to the FPN and the DMN, with overall stable connectivity within the DMN, FPN, and SN. The SMA showed brain plasticity changes, correlating with perinatal depressive and anxiety symptoms. Further studies are needed to better understand the role of brain networks in perinatal neuroplasticity and their contribution to PMAD vulnerability.

## Figures and Tables

**Table 1:** Demographic and clinical measurements.

	N (%)	Mean	SD	Range
Age (years)	22	25.96	3.70	20–32
Education (years)	22	14.52	1.99	12–18
Social support score	22	80.41	6.29	64–88
History of MDD *	0 (0%)			
History of anxiety disorder *	4 (18%)			
Depression score (PHQ-9) at preconception	22	4.36	2.63	0–11
Depression score (PHQ-9) at pregnancy **	22	7.67	3.63	3–15
Depression score (PHQ-9) at postpartum	22	5.18	4.42	1–20
Anxiety score (GAD-7) at preconception	22	2.77	2.64	0–9
Anxiety score (GAD-7) at pregnancy **	22	3.46	2.88	0–10
Anxiety score (GAD-7) at postpartum	22	3.59	4.22	0–18
Pregnancy complications (yes)	6 (27.3%)			
Infant sex (female)	11 (50%)			
Time from enrolling to conception (months)	22	5.27	5.07	0–17
Time from birth to clinical measures (months)	22	1.91	0.75	1–4
Time from birth to postpartum scan (month)	22	5.81	2.94	2–12

* Assessed according to DIAMOND. ** Average score of T1, T2, T3 (three trimesters of pregnancy). MDD = major depressive disorder; PHQ-9 = Patient Health Questionnaire; GAD-7 = General Anxiety Disorder Questionnaire; DIAMOND = Mood and Obsessive Compulsive Disorders and Related Neuropsychiatric Disorders interview.

**Table 2:** Summary of changes in connectivity to the DMN and the FPN from preconception to postpartum.

Network	Direction	Regions	Center of Mass			Mean t *	Size (mm)
			x	z	y		
DMN	Increase	L anterior PFC (10)	−24	54	16	2.56	1846
FPN	Increase	L SMA (6)	−30	10	48	2.50	1671
	Increase	R SMA (6)	25	5	48	2.49	2356
	Decrease	R Ventral PCC (23)	15	−43	37	−2.43	1365

* *p* value < 0.05 for all comparisons. Brodmann region numbering appears in parentheses; DMN = Default-Mode Network; FPN = Frontoparietal Network; PFC = Prefrontal cortex; SMA = Supplementary motor area; PCC = Posterior cingulate cortex; L = left; R = right.

## Data Availability

The data presented in this study are available on reasonable request from the corresponding author. The data is not publicly available due to privacy restrictions.
